# Key H&E morphologic features associated with preoperative misdiagnosis of presacral neoplasms: a retrospective slide re−review study

**DOI:** 10.3389/fonc.2026.1858378

**Published:** 2026-08-11

**Authors:** Heng Deng, Ming Li, Xiaoli Fang, Yingzi Shu, Kun Tang

**Affiliations:** 1 Department of Anorectal Surgery,Second Affiliciated Hospital, Anhui University Of Chinese Medicine, Hefei, China; 2 Department of Anorectal Surgery, The First Affiliated Hospital of Anhui University of Chinese Medicine, Hefei, China; 3 General Practice Department, Anhui Provincial Second People’s Hospital, Hefei, Anhui, China

**Keywords:** H&E staining, pathological morphology, preoperative misdiagnosis, presacral neoplasms, retrospective slide review

## Abstract

Presacral neoplasms are rare retrorectal tumors with highly heterogeneous histogenesis and obscure clinical manifestations. Due to their deep anatomical location and non-specific perianal symptoms, they are frequently misdiagnosed as perianal mimics (benign inflammatory anorectal lesions that mimic presacral tumors clinically), leading to delayed intervention, increased malignant progression risk, and compromised oncological prognosis. The present study aimed to identify core H&E-stained morphological features associated with preoperative misdiagnosis of presacral neoplasms, evaluate their value for lowering preoperative misdiagnosis, and establish a practical pathological diagnostic system for clinical oncological practice. A retrospective diagnostic cohort study was conducted in 112 patients with pathologically confirmed primary presacral neoplasms. H&E sections were independently reviewed by senior pathologists, and logistic regression was applied to identify independent morphological features. The impact of standardized H&E morphological criteria on misdiagnosis rate was quantitatively analyzed. The results identified that three independent H&E morphological features correlated with misdiagnosis risk among presacral neoplasm specimens: specific tumor cell morphology (OR = 6.25, 95% CI:2.48–15.76, P < 0.001), cystic/solid mixed architecture (OR = 5.82, 95% CI:2.31–14.65, P < 0.001), and absence of perianal tissue-specific structures (OR = 5.18, 95% CI:2.05–13.08, P < 0.001). After implementation of the standardized diagnostic criteria, the overall preoperative misdiagnosis rate decreased from 29.5% to 10.2% (P < 0.001), and the biopsy-related misdiagnosis rate dropped from 84.8% to 9.1% (P < 0.001). A representative case of presacral gastrointestinal stromal tumor (GIST) was comprehensively demonstrated with integrated imaging, endoscopic, intraoperative, gross, and H&E pathological features. In conclusion, standardized evaluation based on key H&E morphological features helps lower the preoperative misdiagnosis rate of presacral neoplasms. Standardized interpretation of these indicators significantly reduces misdiagnosis, facilitates early oncological recognition, and improves clinical management efficiency. This strategy is particularly suitable for primary and secondary medical institutions and requires prospective clinical verification.

## 1 Introduction

Presacral (retrorectal) neoplasms represent a heterogeneous group of rare tumors originating from various embryonic remnants, mesenchymal tissues, neurogenic structures (1), and epithelial components in the presacral space (2, 3). Due to the deep and concealed anatomical site, these lesions usually present with non-specific symptoms such as perianal swelling, dull pain, and defecation discomfort, which are highly similar to common benign perianal inflammatory diseases (4). As a result, presacral neoplasms are easily overlooked or misdiagnosed in clinical practice, with reported misdiagnosis rates ranging from 25% to 40% (5). These delays bring multiple adverse clinical consequences for patients (6).

Early-stage presacral neoplasms will progressively expand, invade adjacent organs, and undergo malignant transformation (7, 8). Malignant or borderline subtypes, such as gastrointestinal stromal tumor (GIST) (9), chordoma (10), and neuroendocrine neoplasm (11), carry at high risk of local invasion and distant metastasis without timely intervention. In addition to increasing the difficulty of radical resection, delayed intervention also raises the possibility of irreversible nerve injury, local recurrence, and metastatic spread, which seriously impair long-term oncological prognosis. Preoperative pathological diagnosis is the cornerstone of individualized treatment planning for presacral neoplasms. Among all pathological techniques, hematoxylin-eosin (H&E) staining remains the most fundamental, economical, and widely used method in clinical routine (12), especially in primary hospitals where advanced auxiliary examinations are limited (13). However, in actual clinical practice, pathologists and clinicians often over-interpret inflammatory cell infiltration and ignore characteristic tumor morphological features, resulting in frequent misjudgment of H&E sections.

Current studies mainly focus on imaging features, surgical techniques, or single pathological subtype description of presacral tumors. Few studies have systematically analyzed H&E morphological characteristics linked to preoperative misdiagnosis and no unified morphological evaluation standard has been proposed to lower misdiagnosis risk in routine biopsy assessment. Therefore, this retrospective slide re-review study aimed to identify H&E morphological features associated with preoperative misdiagnosis of presacral neoplasms, quantify whether standardized morphological assessment can reduce misdiagnosis risk, and propose a practical morphological evaluation scheme for pathologists reading presacral biopsy specimens.

## 2 Materials and methods

### 2.1 Study design and population

A retrospective diagnostic cohort study was performed at the Department of Anorectal Surgery, The First Affiliated Hospital of Anhui University of Chinese Medicine. A total of 112 patients with pathologically confirmed primary presacral neoplasms treated between January 2015 and December 2025 were enrolled. This retrospective diagnostic cohort study was reported in accordance with the STROBE (Strengthening the Reporting of Observational Studies in Epidemiology) statement for observational diagnostic research (14, 15).

Inclusion criteria:
Primary presacral neoplasms confirmed by postoperative pathology;
Available preoperative H&E-stained pathological sections;
Complete clinical data including initial diagnosis, misdiagnosis history, imaging, and endoscopic records;
No preoperative antitumor treatment.
Exclusion criteria:
Metastatic tumors or secondary lesions involving the presacral space;
Poor specimen quality or missing H&E images;
Preoperative neoadjuvant chemotherapy, radiotherapy, or targeted therapy;
Incomplete clinical or pathological data.

This study was approved by the Institutional Review Board of The First Affiliated Hospital of Anhui University of Chinese Medicine (IRB No.: AH012-2025). Informed consent was waived, as all clinical and pathological data were fully de-identified before analysis without any personal identifiable information.

### 2.2 Pathological evaluation

H&E-stained sections were independently reviewed by two senior pathologists with more than 10 years of experience in pelvic and perianal pathology. All sections were scanned using an Olympus BX53 light microscope at ×40, ×200, and ×400 magnification.

Evaluated indicators included:
Tumor architecture: cystic, solid, or mixed structure; boundary definition;
Cellular features: specific tumor cell morphology, nuclear atypia, nucleo-cytoplasmic ratio;
Tissue specificity: presence or absence of perianal tissue-specific structures;
Inflammatory pattern: degree and distribution of inflammatory infiltration.

Discrepancies were resolved by a third senior pathologist. Inter-observer consistency was assessed using the kappa test. Inter−observer agreement was substantial (kappa = 0.82), supporting the reliability of morphological evaluation.

Clear binary operational definitions were formulated for the three morphological features:

Specific tumor cell morphology: Atypical neoplastic cells with elevated nucleo-cytoplasmic ratio, nuclear pleomorphism or visible mitoses; pure reactive inflammatory atypia was excluded (score = 1 if present, 0 if absent).
Cystic/solid mixed architecture: Lesions simultaneously contained solid tumor proliferative regions and cystic cavity components; purely solid or purely cystic lesions were scored 0.
Absence of perianal tissue-specific structures: No stratified anal squamous epithelium, anal glands, fistulous granulation tissue or skin adnexa were observed in histological sections (score = 1 if present, 0 if absent).

### 2.3 Diagnostic criteria and outcome measures

All 112 preoperative biopsy H&E slides underwent two separate blind re-reading rounds performed by the same two senior pathologists, with a 3-month washout interval to avoid memory bias. In Round 1 (pre-standardization), pathologists only reviewed clinical history without unified morphological criteria to simulate routine real-world pathological assessment. In Round 2 (post-standardization), pathologists received systematic training on the three morphological indicators and were fully blinded to all preliminary and final postoperative pathological diagnoses, with no cross-reference between two rounds. The primary outcome was the paired preoperative misdiagnosis status of identical slides in two reading rounds. Secondary outcomes included biopsy-related misdiagnosis rate and morphological features correlated with misdiagnosis risk.

### 2.4 Statistical analysis

Statistical analysis was performed using R software (version 4.1.0). Categorical variables were presented as frequencies and percentages. McNemar’s test for paired binary data was adopted to compare misdiagnosis status of identical slides in two blind reading rounds, instead of conventional chi-square test. For morphological risk factors associated with misdiagnosis, univariate logistic regression was first conducted for all candidate histological variables; variables with univariate P < 0.2 entered backward multivariate logistic regression. The Hosmer-Lemeshow goodness-of-fit test was applied to evaluate model calibration, and the sample-to-variable ratio >10 was guaranteed to avoid overfitting. A two-sided P-value < 0.05 was considered statistically significant.

## 3 Results

### 3.1 Baseline characteristics and misdiagnosis status

**Figure 1:**
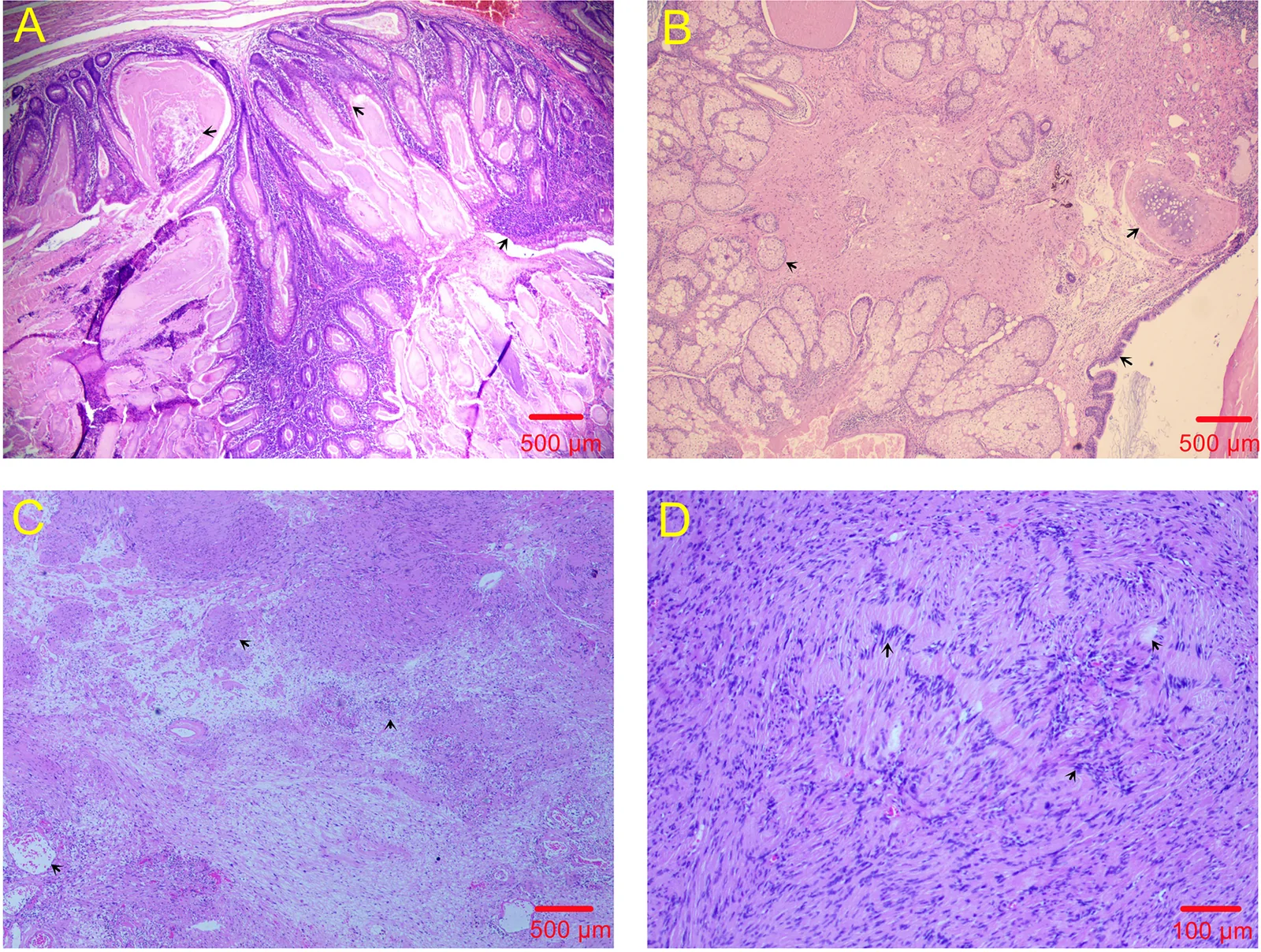
Representative H&E images of common presacral tumors. Representative hematoxylin and eosin (H&E) staining images of common benign presacral tumors. **(A)** Hamartomatous polyp showing typical glandular and stromal components, ×40. **(B)** Teratoma demonstrating mixed tissue elements with distinct structural boundaries, ×40. **(C)** Schwannoma exhibiting uniform spindle cell proliferation with regular architecture, ×40. **(D)** Schwannoma displaying characteristic palisading and fibrillary stroma at higher magnification, ×200. Arrows indicate the three core morphological indicators evaluated in this study.

A total of 112 patients were included. The main initial symptoms were perianal swelling, local pain, and defecation discomfort. Before applying standardized H&E morphological criteria, the overall preoperative misdiagnosis rate was 29.5% (33/112). Among misdiagnosed patients, 84.8% (28/33) had undergone preoperative pathological biopsy, indicating that misinterpretation of H&E morphology was the main cause of misdiagnosis. The most common misdiagnoses were perianal abscess, anal fistula, hidradenitis suppurativa, and benign rectal submucosal tumors (**Figure 1**).

### 3.2 Independent H&E morphological indicators

**Figure 2:**
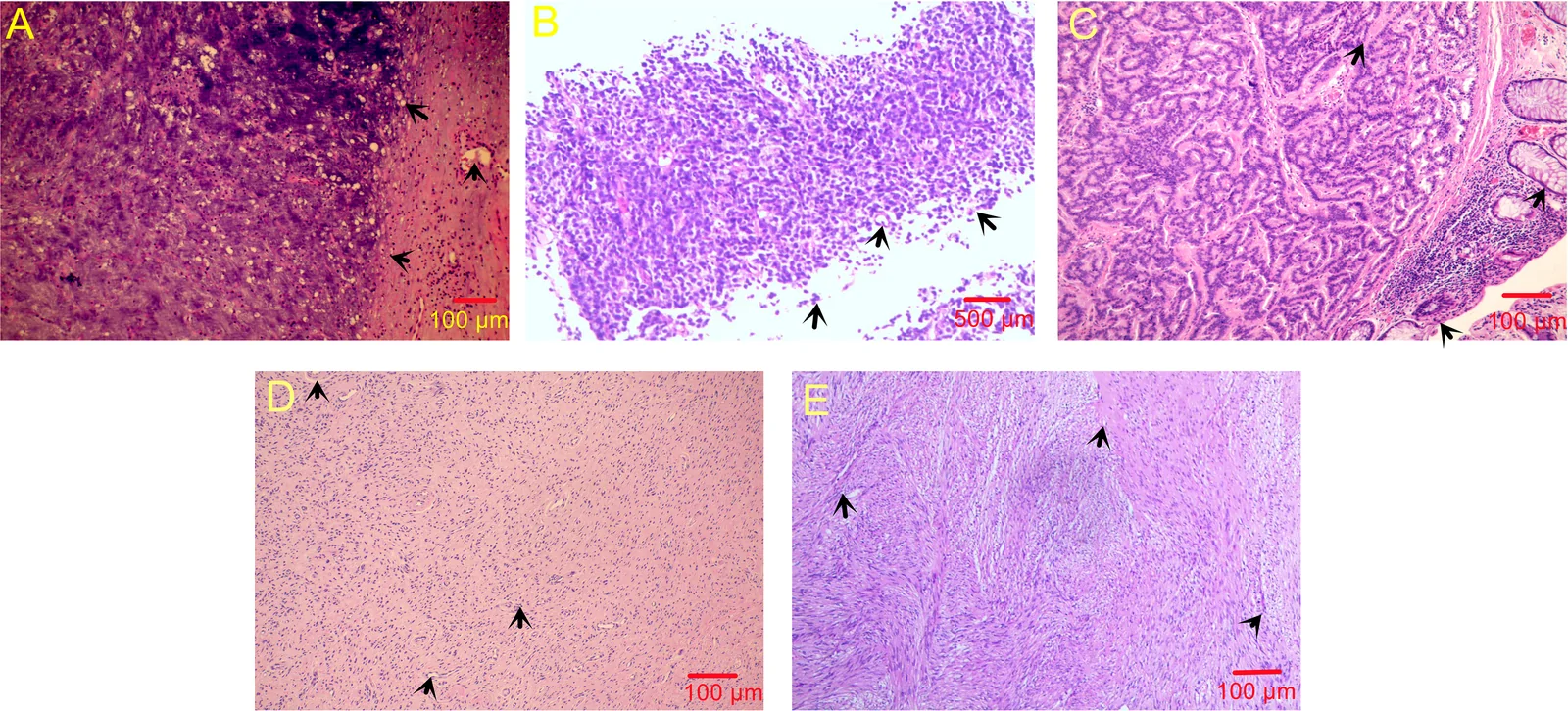
H&E features of rare and malignant presacral tumors. H&E staining images illustrating the morphological characteristics of rare and malignant presacral neoplasms. **(A)** Chordoma showing typical physaliferous cells and lobular growth pattern, ×200. **(B)** Diffuse large B−cell lymphoma presenting diffuse infiltration of large atypical lymphoid cells, ×40. **(C)** Neuroendocrine tumor demonstrating nested growth and monomorphic tumor cells, ×200. **(D)** Neurofibroma showing disordered spindle cell proliferation with myxoid stroma, ×200. **(E)** Gastrointestinal stromal tumor exhibiting spindle−shaped tumor cells with mild−to−moderate atypia, ×200. Arrows indicate the three core morphological indicators evaluated in this study.

All pathologists showed excellent inter-observer consistency (kappa = 0.82). Logistic regression identified three independent morphological features correlated with higher misdiagnosis risk among presacral neoplasm specimens (all P < 0.001):

1. Specific tumor cell morphology (OR = 6.25, 95% CI:2.48–15.76);
2. Cystic/solid mixed architecture (OR = 5.82, 95% CI:2.31–14.65);
3. Absence of perianal tissue-specific structures (OR = 5.18, 95% CI:2.05–13.08) (**Figure 2**) (**Table 1**).

**Table 1:** H&E morphological features correlated with preoperative misdiagnosis of presacral neoplasms.

Indicator	OR	95% CI	P value
Specific tumor cell morphology	6.25	2.48–15.76	< 0.001
Cystic/solid mixed architecture	5.82	2.31–14.65	< 0.001
Absence of perianal tissue−specific structures	5.18	2.05–13.08	< 0.001

Multivariate logistic regression analysis was used to identify independent H&E morphological features associated with misdiagnosis risk within presacral neoplasm samples. OR, odds ratio; CI, confidence interval. All indicators showed high diagnostic value with statistical significance (P < 0.001).

### 3.3 Misdiagnosis reduction after standardized H&E criteria

After applying the standardized H&E morphological diagnostic system, the overall misdiagnosis rate decreased significantly from 29.5% to 10.2% (P < 0.001). Among patients who underwent preoperative biopsy, the misdiagnosis rate dropped from 84.8% to 9.1% (P < 0.001). These results demonstrated that standardized morphological assessment reduces misdiagnosis rates in retrospective blind slide re-evaluation (**Table 2**).

**Table 2:** Misdiagnosis rate of presacral neoplasms before and after application of standardized H&E morphological criteria.

Index	Before	After	P value
Overall misdiagnosis rate	29.5%	10.2%	< 0.001
Biopsy−related misdiagnosis rate	84.8%	9.1%	< 0.001

Comparison of paired misdiagnosis status of identical biopsy slides assessed in two blind reading rounds via McNemar’s test. Standardized interpretation of key H&E features significantly reduced misdiagnosis (P < 0.001).

### 3.4 Integrated diagnostic features of presacral GIST

**Figure 3:**
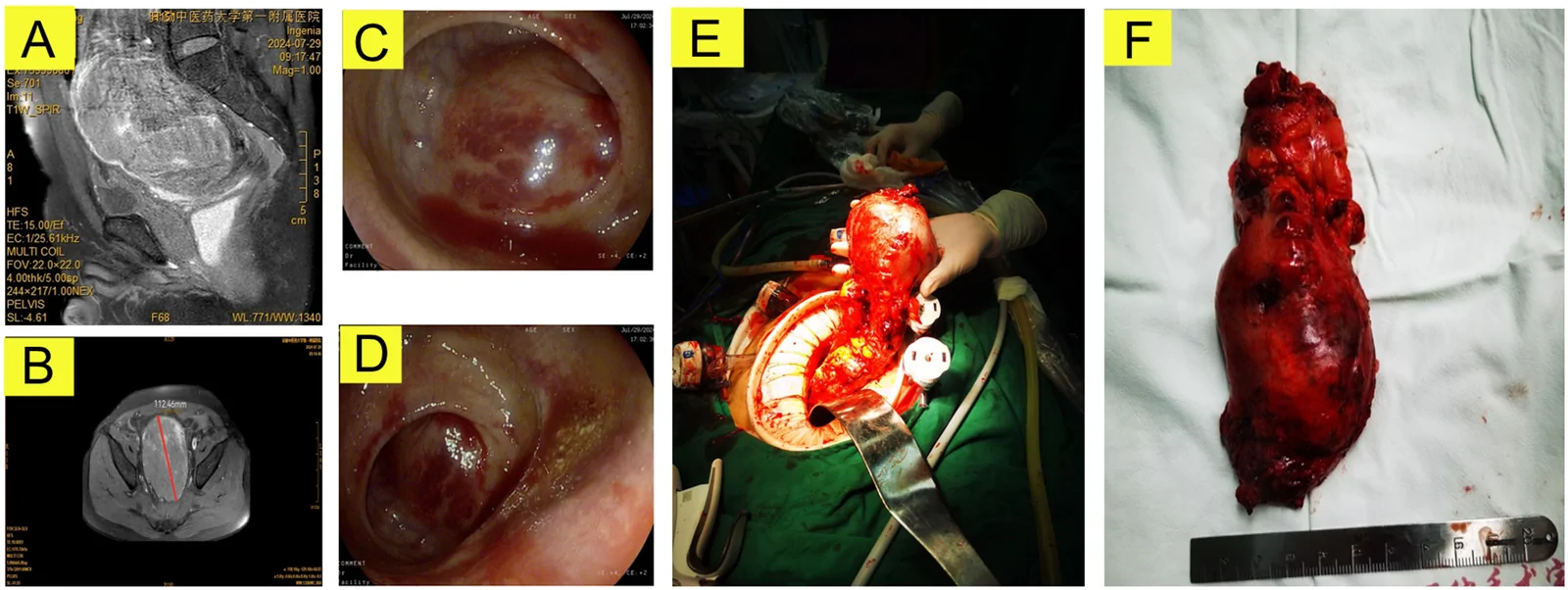
Imaging, endoscopic, intraoperative, and gross features of presacral gastrointestinal stromal tumor (GIST). Comprehensive diagnostic images of presacral GIST. **(A)** Preoperative sagittal MRI showing a well−circumscribed soft−tissue mass in the presacral space. **(B)** Preoperative axial MRI showing homogeneous signal intensity and clear borders of the mass. **(C)** Colonoscopy at 8 cm from the anal verge demonstrating external compression of the posterior rectal wall with intact mucosa. **(D)** Colonoscopy at 5 cm from the anal verge showing mild luminal compression without mucosal erosion or ulceration. **(E)** Intraoperative photograph confirming the tumor located in the presacral space with a clear boundary from the rectal wall. **(F)** Gross specimen showing a solid, well−encapsulated mass with uniform texture.

A typical presacral GIST case was systematically demonstrated:

1. MRI: Well-circumscribed soft-tissue mass in the presacral space with homogeneous signal;
2. Colonoscopy: External compression on the posterior rectal wall without mucosal destruction;
3. Intraoperative finding: Tumor located in the presacral region, separated from the rectal wall;
4. Gross specimen: Solid mass with intact capsule and uniform texture;
5. H&E staining: Spindle-shaped tumor cells with mild-to-moderate atypia, which only provides preliminary morphological hints of mesenchymal lesions on preoperative biopsy. Final pathological confirmation of GIST relies on postoperative immunohistochemistry (CD117, DOG1) and molecular analysis when indicated, though corresponding IHC images are not displayed in this figure. All GIST patients in this cohort completed postoperative IHC testing to reach a definitive diagnosis (**Figure 3**).

## 4 Discussion

Presacral neoplasms are rare and easily misdiagnosed tumors with high clinical heterogeneity (16). Delayed diagnosis is closely associated with increased malignant progression risk and poor oncological prognosis (17). This study summarized three core H&E morphological features related to misdiagnosis risk of presacral neoplasms; standardized assessment of these features reduced the misdiagnosis rate of tumor slides in retrospective blind re-reading.

The three independent H&E morphological features identified in this study reflect histological characteristics that affect pathologists’ judgment of presacral neoplasms during preoperative biopsy reading. Specific tumor cell morphology suggests true neoplastic proliferation rather than inflammatory hyperplasia (18). Cystic/solid mixed architecture is a common feature of congenital and mesenchymal presacral neoplasms, which is rarely seen in simple perianal inflammation (19). Absence of perianal tissue-specific structures helps exclude primary perianal diseases. These features can be easily recognized under conventional light microscopy without additional cost.

The quantitative data confirmed that misdiagnosis was mainly caused by inadequate morphological interpretation rather than lack of pathological examination (20). Overemphasis on inflammatory infiltration often leads to misdiagnosis of neoplasms as abscess or fistula (21). By standardizing the recognition of core tumor morphological features, the overall misdiagnosis rate decreased by two-thirds, and the biopsy-related misdiagnosis rate was almost eliminated.

As a representative mesenchymal neoplasm, presacral GIST is particularly prone to misdiagnosis (22, 23). Our integrated demonstration of imaging, endoscopic, intraoperative, gross, and pathological features provides a comprehensive diagnostic model for clinicians and pathologists. This multi-modal combination helps avoid diagnostic bias caused by single evaluation and improves the accuracy of preoperative oncological assessment. This early identification strategy helps reduce the risk of iatrogenic tumor rupture, unanticipated malignancy, and incomplete resection—all critical for improving oncologic outcomes.

The most prominent advantage of this strategy is its clinical practicability and accessibility. All indicators are derived from routine H&E staining, which requires no immunohistochemistry, molecular testing, or expensive equipment (24). It is highly suitable for widespread application in primary medical institutions, where most patients first visit and misdiagnosis mostly occurs (25). This simple and effective method can bridge the diagnostic gap between primary hospitals and tertiary centers.

This study has several limitations. First, it is a single-center retrospective study with limited number of malignant subtypes, which may lead to potential selection bias. Second, we did not combine H&E features with immunohistochemical or molecular markers for stratified analysis. Third, all diagnostic outcomes came from retrospective slide re-reading by experienced pathologists, which introduces assessment bias compared with real-time clinical biopsy evaluation. Furthermore, we only recruited patients with confirmed presacral neoplasms and lacked a matched perianal inflammatory control cohort; thus, our morphological scheme cannot calculate specificity or distinguish undetermined pelvic lesions, requiring dual-cohort research to validate differential performance. Multicenter prospective studies with larger sample sizes are needed to further verify our findings and develop combined diagnostic models.

## 5 Conclusion

Three key H&E morphological features (specific tumor cell morphology, cystic/solid mixed architecture, and absence of perianal tissue-specific structures) are closely associated with preoperative misdiagnosis risk of presacral neoplasms. Standardized assessment of these morphological features reduces the misdiagnosis rate of tumor specimens in retrospective blind slide re-evaluation and supports more accurate preoperative pathological judgment. This simple, economical, and reproducible strategy requires prospective multicenter verification before wide clinical application and may help optimize clinical management for patients with presacral neoplasms.

## Data Availability

The original contributions presented in the study are included in the article/Supplementary Material. Further inquiries can be directed to the corresponding author.
